# Improving Endothelium-Dependent Vasodilation with Dietary Intake of n-3 Polyunsaturated Fatty Acids-Enriched Chicken Meat: A Randomized Controlled Trial

**DOI:** 10.3390/biomedicines14040852

**Published:** 2026-04-08

**Authors:** Tihana Nađ, Martina Kos, Ana Stupin, Ines Drenjančević, Nikolina Kolobarić, Zrinka Mihaljević, Petar Šušnjara, Mia Damašek, Darjan Kardum, Ivana Jukić

**Affiliations:** 1 Clinic of Pediatrics, University Hospital Centre Osijek, J. Huttlera 4, 31000 Osijek, Croatia; tnad@mefos.hr (T.N.); martina.kos@mefos.hr (M.K.); mdmiadamasek@gmail.com (M.D.); darjan.kardum@mefos.hr (D.K.); 2 Department of Pediatrics, Faculty of Medicine Osijek, Josip Juraj Strossmayer University of Osijek, J. Huttlera 4, 31000 Osijek, Croatia; 3 Department of Physiology and Immunology, Faculty of Medicine Osijek, Josip Juraj Strossmayer University of Osijek, J. Huttlera 4, 31000 Osijek, Croatia; ana.stupin@mefos.hr (A.S.); idrenjancevic@mefos.hr (I.D.); nbdujmusic@mefos.hr (N.K.); zmihaljevic@mefos.hr (Z.M.); 4 Scientific Centre of Excellence for Personalized Health Care, Josip Juraj Strossmayer University of Osijek, Trg Sv. Trojstva 3, 31000 Osijek, Croatia; psusnjara@kifos.hr; 5 Faculty of Kinesiology, Josip Juraj Strossmayer University of Osijek, Drinska 16a, 31000 Osijek, Croatia

**Keywords:** fatty acids, functional food, vascular reactivity, endothelium, microcirculation, flow-mediated dilation

## Abstract

**Objective:** Vascular function serves as an early indicator of cardiovascular (CV) risk. The intake of n-3 polyunsaturated fatty acids (PUFAs) has been reported to improve arterial properties and reduce CV risk, but evidence in healthy individuals remains limited. This study investigated the effects of consuming n-3 PUFAs-enriched chicken meat on vascular reactivity at both microvascular and macrovascular levels in healthy young adults. **Materials and Methods:** In this placebo-controlled, double-blind, randomized interventional trial (ClinicalTrials.gov: NCT05725486), 39 participants (aged 20–26 years) were assigned to either the Control group (n = 20; approximately 118 mg n-3 PUFAs/day) or the n-3 PUFA group (n = 19; approximately 1500 mg n-3 PUFAs/day) for three weeks. Microvascular reactivity was assessed via post-occlusive reactive hyperemia (PORH), acetylcholine-induced dilation (AChID), local thermal hyperemia (LTH), and sodium nitroprusside-induced (SNPID) responses. Macrovascular reactivity was measured by brachial artery flow-mediated dilation (FMD) and nitroglycerine-mediated dilation (NTG-MD). Body composition and blood pressure (BP) were recorded before and after the intervention. **Results:** Both microvascular (PORH, AChID, and LTH) and macrovascular (FMD) endothelium-dependent vasodilation increased in the n-3 PUFAs group following the dietary protocol compared to the Control group. Conversely, the three-week dietary intervention did not influence endothelium-independent dilation in either the microvasculature (SNPID) or macrovasculature (NTG-MD) within the groups compared to baseline, nor were any differences observed between the groups. No significant changes were noted in BP or body composition after either diet. **Conclusions:** In healthy young adults, consuming the n-3 PUFAs-enriched chicken meat for three weeks improved endothelium-dependent vasodilation in both micro- and macrocirculation, without affecting endothelium-independent responses. These findings suggest that dietary n-3 PUFA intake may provide vascular benefits even in healthy, disease-free individuals at rest.

## 1. Introduction

Globally, cardiovascular (CV) diseases remain the predominant cause of morbidity and mortality, placing a substantial burden on healthcare systems and society. Atherosclerosis and its associated risk factors—such as hypertension, dyslipidemia, obesity, and chronic low-grade inflammation—are recognized as central drivers in the development and progression of CV diseases [1,2]. Accumulating evidence indicates that vascular dysfunction, particularly endothelial dysfunction, often precedes overt structural changes in the arterial wall and can serve as an early and sensitive marker of CV risk [3].

Endothelial dysfunction is commonly described as impaired endothelium-dependent vasodilation accompanied by reduced nitric oxide (NO) bioavailability, increased reactive oxygen species (ROS) production, and vascular inflammation, all of which contribute to the initiation and progression of atherosclerotic lesions and other vascular pathologies [3]. Importantly, endothelial dysfunction is now considered a systemic process affecting both conduit arteries and the microcirculation, underscoring its central role in overall cardiovascular health and prevention strategies.

Lifestyle-based strategies, including dietary modification and regular physical activity, are recognized as fundamental approaches to preventing CV disease and promoting overall vascular health [4]. In recent decades, functional foods have assumed an increasingly prominent role in nutrition and disease prevention. Functional foods are defined as foods that, beyond their basic nutritional value, contain biologically active components capable of modulating physiological functions and contributing to disease risk reduction; they may be either naturally occurring or intentionally modified to enhance their health-promoting properties [5]. Among dietary components, long-chain omega-3 polyunsaturated fatty acids (n-3 PUFAs), primarily eicosapentaenoic acid (EPA) and docosahexaenoic acid (DHA), have attracted considerable scientific interest due to their multiple vasoprotective, anti-inflammatory, and metabolic effects, which include improving endothelial function, reducing oxidative stress, and modulating lipid and glucose metabolism [6,7,8]. Traditionally, oily fish have been the primary dietary source of n-3 PUFAs, but consumption is often limited by availability, taste preferences, or cultural dietary habits. In this context, functional foods enriched with these fatty acids—such as hen eggs or poultry meat—have emerged as a practical and accessible alternative, providing an effective means to increase n-3 PUFA intake, particularly in populations with low fish consumption or in individuals seeking convenient dietary options [4].

Evidence from clinical and experimental studies demonstrates that supplementation with n-3 PUFAs improves arterial compliance, lowers blood triglyceride levels, and modulates inflammatory and oxidative pathways, thereby reducing CV risk in individuals with established CV disease or metabolic syndrome [6,9]. Mechanistically, these effects are mediated through the incorporation of EPA and DHA into cell membrane phospholipids, leading to the generation of specialized pro-resolving lipid mediators, including resolvins, protectins, and maresins, which collectively support vascular homeostasis [10]. Despite these well-established benefits in at-risk populations, evidence concerning the impact of n-3 PUFA intake on vascular function in young, otherwise healthy individuals remains limited. Our research group conducted a series of randomized, double-blind, placebo-controlled trials in healthy young adults showing that n-3 PUFA-enriched functional foods can beneficially modulate vascular and systemic health [7,8,11]. These effects are mediated by the modulation of endothelium-derived vasoactive mediators (e.g., NO, COX- and CYP450-derived metabolites), attenuation of oxidative stress, suppression of endothelial activation, regulation of leukocyte–endothelium interactions, and subsequent reduction in vascular inflammation [12,13]. In our previous work, we demonstrated that higher-dose-enriched eggs (~1 g/day) improved microvascular endothelial function and reduced pro-inflammatory markers, while a lower dose (~0.4 g/day) did not enhance vasoreactivity but still influenced leukocyte activation and redox balance [8,11]. Enriched chicken meat (~1.5 g/day) further demonstrated robust anti-inflammatory, antioxidative, and pro-resolving effects, highlighting functional foods as a practical strategy to deliver biologically active n-3 PUFAs even in low-risk populations [7]. Although daily consumption of n-3 PUFAs has been shown to improve microvascular endothelium-dependent reactivity in young healthy adults [8], likely by modulating the balance of pro- and anti-inflammatory factors, the potential vascular effects of n-3 PUFAs provided via functional foods, such as enriched chicken meat, remain unexplored.

This randomized trial aimed to investigate whether daily consumption of chicken meat naturally enriched with n-3 PUFAs improves vascular function in healthy young adults. Specifically, we assessed the effects of three weeks of enriched chicken meat intake on both endothelium-dependent and vascular smooth muscle-dependent vasodilation, at the microvascular and macrovascular levels, representing the first randomized controlled evaluation of n-3 PUFA delivery through an enriched chicken meat matrix. Given that the absorption, bioavailability, and metabolic effects of n-3 PUFAs may vary across food vehicles, demonstrating vascular effects in this distinct functional food matrix extends previous findings and supports translational nutrition strategies. We hypothesized that increasing dietary n-3 PUFAs intake through functional food–based delivery would enhance endothelial function, as reflected by improved microvascular reactivity and brachial artery flow-mediated dilation, even in individuals without overt CV disease risk.

## 2. Materials and Methods

### 2.1. Study Participants and Protocol

Forty healthy young adults (20 women and 20 men), aged between 20 and 26 years, were recruited in this randomized, double-blind, placebo-controlled study (registered at clinicaltrials.gov, NCT05725486), although one participant withdrew for personal reasons. Thus, a total of 39 healthy volunteers completed the study. Exclusion criteria included a history of smoking, hypertension, diabetes, dyslipidemia, chronic inflammatory conditions, cerebrovascular or renal disease, coronary or peripheral artery disease, as well as the use of any medications or substances known to influence endothelial function. None of the participants consumed dietary supplements or functional foods (including, but not limited to, n-3 PUFAs) before or during the study period.

All participants took part voluntarily, and no financial or other compensation was offered for their involvement in the study. The study protocol was explained in detail to all subjects, and written informed consent was obtained from each subject. The study protocol and procedures conformed to the latest revision of the Declaration of Helsinki and were approved by the Ethical Committee of the Faculty of Medicine, University of Osijek (Cl: 602-04/23-08/03; No.: 2158-61-46-23-125).

**Figure 1:**
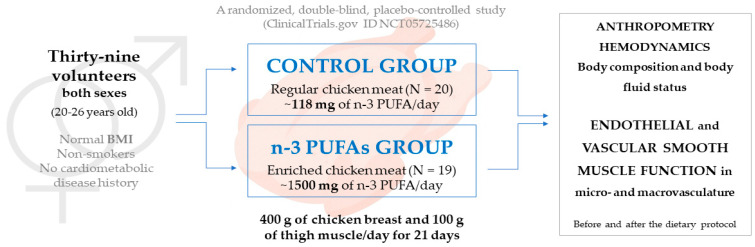
Study design. BMI—body mass index; n-3 PUFAs—n-3 polyunsaturated fatty acids.

The subjects were divided into two groups by a simple randomization procedure using a coin-toss procedure by an independent investigator not involved in recruitment, outcome assessment, or data analysis, while both participants and outcome assessors were blinded to group allocation through identical preparation and distribution of intervention products. A total of 20 subjects comprised the Control group (W/M = 12/8), which consumed regular chicken meat (breast and thigh muscle, n-3 PUFAs content ~118 mg/day), and 19 subjects comprised the n-3 PUFAs group (W/M = 8/11), which consumed n-3 PUFA-enriched chicken meat (breast and thigh muscle, n-3 PUFAs content ~1500 mg/day). The dietary protocol of the study lasted for 3 weeks, during which the participants had two study visits. On the first study day, following baseline assessments, each participant received the full quantity of prepackaged meat required for the protocol. Each daily portion consisted of 400 g of chicken breast and 100 g of chicken thigh. Participants were instructed to consume one package per day, prepared either by boiling or brief searing in a small amount of olive oil. The amount of chicken consumed in this study was relatively high and may not fully reflect usual dietary habits; however, the intervention was designed to ensure controlled and consistent intake of n-3 PUFAs over a short period. Participant compliance was monitored through daily dietary records and controlled distribution of pre-portioned meat packages. Participants were instructed to maintain their usual diet, consume one portion per day, and avoid any additional meat intake or n-3 PUFA supplementation. The follow-up visit occurred the day after completion of the intervention, during which all measurements were repeated. The study was carried out in the Laboratory for Clinical and Sports Physiology, Department of Physiology and Immunology, Faculty of Medicine, University of Osijek, Osijek, Croatia. Figure 1 shows the study design.

The full study protocol, including the CONSORT diagram and details on enriched meat production, has been published previously in Nađ et al. (2025) [7]. Therefore, only a concise overview is provided here, as the present analysis refers to the same cohort of study participants described in that publication. Importantly, the fatty acid composition of both the n-3 PUFAs-enriched and regular chicken meat used in this study has been previously characterized [6]. The enriched chicken meat contained a significantly higher proportion of n-3 PUFAs and a markedly lower n-6/n-3 ratio compared with regular chicken meat. Accordingly, in the present trial, the two intervention groups differed primarily in n-3 PUFAs intake, while the quantity, preparation method, and overall macronutrient composition of the consumed meat were comparable.

### 2.2. Body Mass Index and Arterial Blood Pressure Measurements

Body weight (kg) was measured using a personal scale (Radwag, Radom, Poland) with participants wearing light clothing and no shoes. Height (m) was also recorded, and body mass index (BMI) was calculated using the formula: weight/height^2^ (kg/m^2^) [14]. Waist and hip circumferences (cm) were measured to determine the waist-to-hip ratio (WHR).

Arterial blood pressure and heart rate (HR) were measured using an automated oscillometric sphygmomanometer (OMRON M3, OMRON Healthcare Inc., Osaka, Japan) following a 15 min rest in a seated position. The final values for blood pressure and HR were obtained by averaging three consecutive readings. Mean arterial pressure (MAP) was calculated using the formula: MAP = [SBP + 2(DBP)]/3 [15].

### 2.3. Assessment of Body Composition and Body Fluid Status

Body composition and fluid status were assessed using a 4-terminal portable bioelectrical impedance analyzer (Maltron Bioscan 920-II, Maltron International Ltd., Rayleigh, Essex, UK).

Measurements were conducted with participants in a supine position, arms positioned alongside the body but not touching the trunk, and legs slightly apart. Four sensing electrodes were applied—two on the dorsal side of the wrist and two on the anterior surface of the ankle—to enable whole-body impedance analysis. Using empirically derived formulas, the manufacturer’s original software generated data on the proportion of muscle mass, fat-free mass, fat mass, total body water, extracellular water (ECW), intracellular water (ICW), plasma volume, interstitial fluid volume, and body density.

### 2.4. Assessment of Microvascular Endothelial Function—Laser Doppler Flowmetry of Peripheral Skin Microcirculation

Microvascular responses in the forearm skin were assessed using the laser Doppler flowmetry (LDF) technique (MoorVMS-LDF, Axminster, UK), following previously established protocols from our laboratory [16]. Endothelium-dependent vasorelaxation was evaluated by analyzing the microvascular responses to various stimuli: post-occlusive reactive hyperemia (PORH), iontophoretically applied acetylcholine (acetylcholine-induced dilation, AChID), and local skin heating (local thermal hyperemia, LTH). To assess endothelium-independent vasorelaxation, iontophoretically applied sodium nitroprusside was used (sodium nitroprusside-induced dilation, SNPID). Acetylcholine (1%) and sodium nitroprusside (1%) were administered using standardized pulsed iontophoresis protocols after a 5 min baseline recording. ACh was delivered as seven pulses of 0.1 mA for 30 s (30 s intervals), whereas SNP was delivered as three pulses of 0.1 mA followed by four pulses of 0.2 mA for 30 s (90 s intervals). Blood flow was measured in arbitrary perfusion units (PU). PORH was determined by calculating the difference in blood flux between reperfusion and occlusion, expressed as a percentage change (R-O%), relative to the initial baseline value. AChID and SNPID were measured as the increase in perfusion after iontophoresis of acetylcholine or sodium nitroprusside, compared to basal values before the substances were applied. LTH reflects the increase in blood flow in response to local skin heating [16].

### 2.5. Assessment of Macrovascular Endothelial Function—Flow-Mediated Dilation (FMD) of the Brachial Artery

The FMD technique was used to assess arterial function in vivo as the percentage dilation of the brachial artery after a period of forearm occlusion. Initially, the baseline diameter and blood flow velocity of the right brachial artery were imaged using an ultrasound vascular probe (Vivid iq, GE Healthcare, Chicago, IL, USA). After recording the baseline flow velocity, a blood pressure cuff (placed distal to the imaged artery) was inflated to 60 mmHg above the baseline systolic BP for 5 min, and the artery was then continuously imaged following the release of the BP cuff. The response to NTG was used to determine endothelium-independent vasodilation. A single sublingual NTG spray (0.4 mg) was administered after obtaining the baseline brachial artery diameter, and brachial artery images and measurements were repeated for the following 5 min. Digital images were acquired with Brachial Imagery (Medical Imaging Applications, Iowa City, IA, USA) and analyzed as previously reported [17].

### 2.6. Statistical Analysis

An a priori sample size calculation was performed based on the primary outcome measure. Effect size was estimated using Cohen’s d (Δ/SD) derived from pilot data obtained from 16 participants during the study preparation phase. Based on the estimated effect size, a two-tailed significance level of 0.05, and a statistical power of 80%, the required sample size was calculated to be 13 participants per group.

The results were reported as mean and standard deviation (SD). The normality of the distribution of numerical variables was determined by the Kolmogorov–Smirnov normality test. To assess the differences within groups (measurements before and after the study protocol), the Wilcoxon rank-sum test was applied for variables that were not normally distributed, while the paired *t*-test was used for normally distributed data. Differences between groups in measurements at the end of the protocol were evaluated using analysis of covariance (ANCOVA), with adjustments made for baseline (pre-measurement) values. *p* < 0.05 was considered statistically significant. For statistical analysis, SigmaPlot version 15 (Systat Software, Inc., Chicago, IL, USA) was used, and all graphs were generated using GraphPad Prism 6 (GraphPad Software, Inc., San Diego, CA, USA).

## 3. Results

### 3.1. Anthropometric and Blood Pressure Measurements

Anthropometric and hemodynamic parameters of study participants are presented in Table 1. Participants represented a generally healthy young cohort, with no cases of obesity. All participants had normal systolic BP, diastolic BP, and MAP values and were considered normotensive. There was no statistically significant difference in all measured parameters (age, BMI, WHR, SBP, DBP, MAP, and HR values) after three weeks of consumption of regular or n-3 PUFAs-enriched chicken meat compared to baseline (initial) measurements. Furthermore, there was no statistically significant difference in all measured parameters between the examined groups.

### 3.2. Body Composition and Body Fluid Status

Table 2 summarizes the impact of the dietary protocols on the body composition and body fluid status of the participants. There were no significant differences in all measured parameters: fat-free mass (FFM %), fat %, total body water (TBW %), extracellular water (ECW %), intracellular water (ICW %), plasma fluid (PF), interstitial fluid (IF), or body density before and after dietary protocol within the Control and n-3 PUFAs group. There were no significant differences in all measured parameters between the examined groups, neither before nor after the respective study protocols.

### 3.3. Peripheral Skin Microvascular Endothelium-(In)Dependent Dilator Function

**Figure 2:**
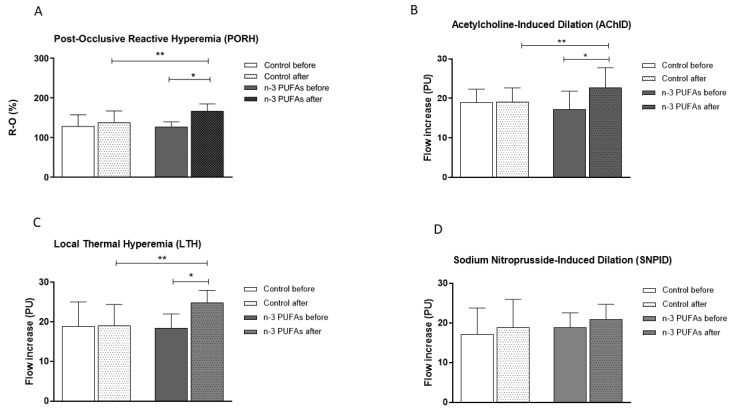
The effect of three-week regular (Control group) and n-3 PUFAs-enriched (n-3 PUFAs group) chicken meat dietary intake on endothelium-dependent and endothelium-independent skin peripheral microvascular reactivity: post-occlusive reactive hyperemia (PORH) (**A**); response to iontophoretically applied acetylcholine (AChID) (**B**), local thermal hyperemia (LTH) (**C**), and response to iontophoresis of sodium nitroprusside (SNP) (**D**). Results are expressed as mean and standard deviation (SD). *p* * < 0.05 before vs. after within the n-3 PUFAs group—paired *t*-test; *p* ** < 0.05 Control vs. n-3 PUFAs group—analysis of covariance (ANCOVA). Flow increase represents the absolute change in perfusion between baseline and stimulus values. PU—perfusion units.

Consumption of n-3 PUFAs-enriched chicken meat significantly improved forearm skin microvascular endothelium-dependent vasodilation in responses to vascular occlusion (PORH, Figure 2A), iontophoretically applied acetylcholine (AChID, Figure 2B), and to local skin heating (LTH, Figure 2C) compared to baseline measurement within the n-3 PUFAs group. In contrast, consumption of regular chicken meat did not induce any significant change in endothelium-dependent responses of forearm skin microcirculation compared to baseline in the Control group (Figure 2A–C). Furthermore, there were statistically significant differences in PORH, AChID, and LTH between the examined groups (Figure 2A–C). On the other side, consumption of regular or n-3 PUFAs-enriched chicken meat did not affect the endothelium-independent microvascular response to iontophoretically applied SNP; SNPID was similar before and after a three-week daily consumption of regular or enriched chicken meat within the Control or n-3 PUFAs group, and similar between the examined groups (Figure 2D).

### 3.4. Peripheral Macrovascular Endothelium-(in)Dependent Dilator Function

**Figure 3:**
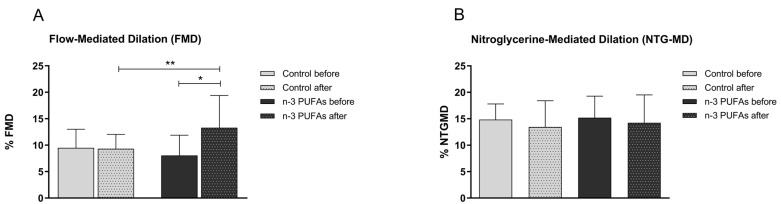
The effect of three-week regular (Control group) and n-3 PUFAs-enriched (n-3 PUFAs group) chicken meat dietary intake on brachial artery flow-mediated dilation, FMD (**A**), and nitroglycerine-mediated dilation, NTG-MD (**B**). Results are expressed as mean and standard deviation (SD). *p* * < 0.05 before vs. after within the n-3 PUFAs group—paired *t*-test; *p* ** < 0.05 Control vs. n-3 PUFAs group—analysis of covariance (ANCOVA).

Macrovascular endothelial vasodilation assessed by flow-mediated dilation of the brachial artery was significantly increased following consumption of n-3 PUFAs-enriched chicken meat for three weeks, but not regular chicken meat, compared to baseline measurements (Figure 3A). FMD was significantly higher in the n-3 PUFAs group compared to the Control group after the study protocol (Figure 3A). On the other side, consumption of regular or n-3 PUFAs-enriched chicken meat did not affect the endothelium-independent response of the brachial artery to nitroglycerine; after a three-week consumption of regular or n-3 PUFAs-enriched chicken meat, NTG-MD was unchanged compared to baseline values, and no significant difference was observed between the examined groups (Figure 3B).

## 4. Discussion

The current scarcity of randomized clinical trials, particularly those investigating functional foods, highlights the need for increased attention from both the scientific community and the public. Further well-designed clinical studies are needed to better define the health effects of individual bioactive compounds and to clarify their potential role in preventive healthcare. In this context, functional foods enriched with n-3 PUFAs provide an innovative and practical dietary approach to increase daily n-3 PUFA intake without necessitating substantial changes in eating habits. Incorporating these fatty acids into commonly consumed food products may represent an effective strategy for promoting early vascular health, even among individuals without apparent cardiovascular risk factors. This is the first randomized, double-blind, placebo-controlled interventional study to investigate the effects of consuming n-3 PUFAs-enriched chicken meat on vascular reactivity at both microvascular and macrovascular levels in young, healthy individuals. These are the key findings of this study: (a) in the group that consumed n-3 PUFAs-enriched chicken meat, both micro- (PORH, AChID, LTH) and macrovascular (FMD) endothelium-dependent vasodilation was significantly increased compared to baseline and compared to controls; (b) the three-week dietary protocol did not affect endothelium-independent dilation in either the microvasculature (SNPID) or macrovasculature (NTG-MD) within both examined groups compared to baseline values, nor were any differences observed between the groups; (c) consumption of regular or fortified meat did not affect blood pressure or body composition.

Over the past few decades, PUFAs have gained increasing attention for their importance in human health. Previously, it was suggested that higher intake of n-3 PUFAs reduces CV risk, as n-3 PUFAs exert antioxidant, anti-inflammatory, and antithrombotic effects, improve glucose and lipid metabolism, stabilize cardiac electrophysiology, and positively influence vascular function and BP regulation [18]. Epidemiological and experimental evidence suggest that n-3 PUFAs, particularly DHA and EPA, may confer CV protection by modulating modifiable risk factors. For instance, EPA consumption was linked to a lower incidence of major vascular events in comprehensive and very significant interventional studies [19,20]. The main effects of n-3 PUFAs supplementation are most pronounced in CV patients, e.g., n-3 PUFAs may lead to a clinically meaningful reduction in BP among individuals with untreated hypertension [21], those with essential hypertension [22,23], and mildly hypercholesterolemic yet normotensive subjects [24]. However, a comparable blood pressure-lowering effect of n-3 PUFAs has not been demonstrated in normotensive individuals [25], nor was it observed in the current study. Furthermore, in our previous study, which was conducted on healthy young people who ingested ~1053 mg of n-3 PUFAs/day for three weeks in the form of enriched hen eggs, no effect on BP values was observed [26]. Nevertheless, we demonstrated that young healthy individuals who consumed 777 mg of n-3 PUFAs/day in the form of enriched hen eggs for three weeks had reduced BP [26], consistent with the findings of Oh et al. [27]. Interestingly, a notable decrease in BP was also observed in healthy participants who consumed three regular hen eggs over the same period. Therefore, the observed reduction in BP cannot be attributed solely to n-3 PUFA intake.

The influence of n-3 PUFA intake on body composition remains unclear. Current clinical evidence suggests that dietary intake and supplementation with n-3 PUFAs have minimal or non-significant effects on body composition and body fluid status in healthy, lean individuals, overweight adults, and obese adults. Systematic reviews and meta-analyses of randomized controlled trials consistently show that n-3 PUFA supplementation, whether from fish, fish oil, or enriched eggs, does not produce clinically meaningful reductions in body weight, fat mass, or lean mass in these populations [28,29,30]. Consistently, in the present study, we did not observe any significant changes in measured body composition parameters or body fluid status following the consumption of n-3 PUFAs-enriched chicken meat in healthy, lean individuals. There is a lack of direct evidence regarding the impact of n-3 PUFAs intake on body fluid status in humans, as this outcome is rarely measured in clinical trials or reviews [31,32]. Overall, the clinical utility of n-3 PUFA supplementation for body composition is supported by mechanistic and interventional data, but the magnitude of effect is modest and highly variable across individuals. No guideline from a major professional society currently recommends omega-3 PUFAs specifically for body composition or body fluid management.

The intake of n-3 PUFAs has been shown to exert beneficial effects on vascular function, particularly by enhancing endothelial-dependent vasodilation in both healthy individuals [33,34] and those with CV risk factors [35,36]. The magnitude and clinical relevance of these effects are influenced by population characteristics and the specific n-3 PUFA formulation used [13,36,37,38,39]. In our earlier randomized study involving healthy young adults, we have shown that daily consumption of n-3 PUFAs-enriched hen eggs (providing approximately 777 mg or 1053 mg n-3 PUFAs per day for three weeks) resulted in significant improvements in skin microvascular endothelium-dependent vasodilation, as assessed by PORH and iontophoretic ACh application. Notably, n-3 PUFA intake did not significantly affect endothelium-independent vasodilatory responses, such as those elicited by SNP [26]. The absence of changes in endothelium-independent vasodilation, alongside improvements in endothelium-dependent responses, suggests that the observed effects are primarily mediated through enhanced endothelial function rather than alterations in vascular smooth muscle responsiveness. This study is the first to show that n-3 PUFAs intake enhances peripheral microvascular endothelium-dependent responses to local thermal heating in young healthy adults (Figure 2C). We also confirmed that n-3 PUFAs improve endothelial function (Figure 2A,B) even when consumed through enriched chicken meat. In line with our previous findings, n-3 PUFAs did not influence endothelium-independent microvascular response (Figure 2D). Except in the microcirculation, recent meta-analyses and interventional studies indicate that n-3 PUFAs promote improvements in macrovascular health by enhancing endothelial function, decreasing arterial stiffness, and increasing nitric oxide bioavailability. These vascular benefits appear to be more pronounced in clinical populations compared with healthy individuals. Evidence also suggests a dose-dependent effect, with intakes exceeding 1 g/day providing greater benefits than lower doses [36,38,40]. Our study also supports this finding. Specifically, macrovascular endothelium-dependent vasodilation, but not endothelial-independent vasodilation, was significantly improved in response to n-3 PUFAs-enriched chicken meat consumption in young healthy individuals. In this context, the observed increase in FMD reflects improved endothelial function, an early marker of vascular health and a predictor of future CV risk. In healthy individuals, such improvement does not indicate treatment of existing disease but rather suggests a potential protective effect by supporting vascular homeostasis and delaying the development of endothelial dysfunction. Within the same cohort of participants, we have previously demonstrated that n-3 PUFAs can beneficially modulate physiological processes related to oxidative balance [7]. In that study, consumption of n-3 PUFAs-enriched chicken meat was associated with a significant reduction in systemic inflammation, reflected by decreased hsCRP levels, along with enhanced antioxidant capacity (increased FRAP), increased activity of antioxidant enzymes (GPx and SOD), and reduced intracellular production of reactive oxygen species in peripheral blood mononuclear cells. Furthermore, levels of specialized pro-resolving lipid mediators, including resolvin E1 and resolvin D1, were significantly increased. These findings suggest that the improvement in endothelium-dependent vasodilation observed in the present study may be mediated, at least in part, by reduced oxidative stress and inflammation, as well as enhanced pro-resolving signaling pathways. Together, these findings provide complementary functional and biochemical evidence supporting the vascular benefits of n-3 PUFAs-enriched functional food. These findings further support anti-inflammatory effects observed in studies by other researchers, and together with their antithrombotic and antioxidant effects [7,11,41], n-3 PUFAs raise their practical implications in the context of cardiovascular disease prevention and health promotion.

The present study provides a translational perspective by demonstrating that n-3 PUFAs-enriched chicken meat, a widely consumed and culturally acceptable functional food, can serve as an effective dietary vehicle for increasing n-3 PUFA intake while improving vascular function. While this may appear incremental from a purely mechanistic perspective, the food matrix, potential differences in bioavailability, compliance profile, and real-world applicability of dietary delivery strategies are important considerations in preventive cardiovascular nutrition. Consistent vascular benefits across different food sources further support the practical relevance of n-3 PUFA interventions. This real-world dietary approach can be implemented without major changes in eating habits and may support the early maintenance of endothelial function and vascular health under controlled conditions. While these findings suggest a potential role of n-3 PUFAs in supporting vascular homeostasis, they should be interpreted as short-term physiological effects rather than direct evidence of clinically meaningful cardiovascular protection.

Our study has some limitations, including the relatively short intervention period. While a minimum duration of one month is generally recommended, our study employed a three-week dietary protocol. Nevertheless, despite this brief intervention, our results demonstrated an improvement in endothelium-dependent vasodilation in macrocirculation and microcirculation with the ingestion of n-3 PUFAs-enriched meat. Furthermore, although this study included only young, healthy individuals, this may also be considered a strength, as most consistent evidence for the beneficial effects of n-3 PUFAs comes from CV patients, whereas their impact in generally healthy populations remains less well studied.

## 5. Conclusions

In conclusion, this study demonstrates that n-3 PUFAs from functional food-enriched chicken meat improve both microvascular and macrovascular endothelium-dependent vasodilation in healthy young subjects, without affecting endothelium-independent vasodilation. These effects occur independently of changes in blood pressure, body composition, or fluid status. The findings should be interpreted as short-term physiological improvements in endothelial function, a recognized early marker of cardiovascular health. Further studies with larger sample sizes, longer intervention duration, and clinical endpoints are needed to determine their long-term clinical relevance.

## Figures and Tables

**Table 1:** The effect of regular (Control group) and n-3 PUFAs-enriched meat (n-3 PUFAs group) consumption on anthropometric and hemodynamic parameters.

Parameter	Control Group			n-3 PUFAs Group			*p* ^‡^
N (W/M)	20 (12/8)			19 (8/11)			0.527
Age (years)	23 (3)			23 (3)			0.476
	Before	After	*p* ^†^	Before	After	*p* ^†^	
BMI (kg/m^2^)	24.8 (4.9)	24.6 (5.0)	0.978	23.9 (3.1)	24.0 (3.1)	0.871	0.366
WHR	0.81 (0.05)	0.82 (0.05)	0.813	0.82 (0.05)	0.82 (0.05)	0.854	0.975
SBP (mmHg)	117 (12)	114 (11)	0.09	116 (11)	113 (9)	0.097	0.98
DBP (mmHg)	77 (9)	77 (8)	0.764	73 (6)	74 (6)	0.575	0.749
MAP (mmHg)	91 (9)	89 (8)	0.312	88 (7)	86 (7)	0.953	0.56
HR (beats per min)	73 (10)	72 (10)	0.694	75 (12)	75 (15)	0.99	0.652

Results are expressed as mean and standard deviation (SD). N—number of participants; BMI—body mass index; SBP—systolic blood pressure; DBP—diastolic blood pressure; MAP—mean arterial pressure; HR—heart rate. *p* ^†^ before vs. after within the group (Control or n-3 PUFAs)—paired *t*-test; *p* ^‡^ between groups with adjustments for baseline (analysis of covariance (ANCOVA)).

**Table 2:** The effect of regular (Control group) and n-3 PUFAs-enriched meat (n-3 PUFAs group) consumption on body composition and body fluid status.

Parameter	Control Group			n-3 PUFAs Group			*p* ^‡^
	Before	After	*p* ^†^	Before	After	*p* ^†^	
Fat-Free Mass (%)	75.8 (9.1)	73.9 (16.9)	0.807	80.9 (8.0)	82.7 (6.6)	0.226	0.299
Fat (%)	24.2 (9.1)	24.1 (10.7)	0.84	19.1 (8.0)	17.3 (6.6)	0.227	0.174
Total Body Water (%)	57.5 (8.3)	59.5 (8.3)	0.309	61.5 (8.0)	64.3 (7.2)	0.139	0.24
Extracellular Water (%)	44.2 (1.4)	44.4 (1.6)	0.632	44.5 (2.5)	42.7 (10.0)	0.78	0.475
Intracellular Water (%)	55.8 (1.4)	55.5 (1.8)	0.758	55.5 (2.5)	55.2 (3.8)	0.89	0.824
Plasma Fluid (L)	4.0 (1.0)	4.1 (0.8)	0.502	4.2 (0.)	4.5 (1.2)	0.393	0.278
Interstitial Fluid (L)	13.8 (3.4)	14.2 (2.8)	0.488	14.7 (2.9)	15.8 (4.3)	0.393	0.278
Body Density (kg/L)	1.044 (0.021)	1.048 (0.02)	0.22	1.055 (0.018)	1.06 (0.015)	0.583	0.312

Results are expressed as mean and standard deviation (SD). *p* ^†^ before vs. after within the group (Control or n-3 PUFAs)—paired *t*-test; *p* ^‡^ between groups with adjustments for baseline (analysis of covariance (ANCOVA)).

## Data Availability

The original contributions presented in this study are included in the article; further inquiries can be directed to the corresponding author.

## Abbreviations

The following abbreviations are used in this manuscript:

AChID	acetylcholine-induced
BMI	body mass index
BP	blood pressure
CV	cardiovascular
CVD	cardiovascular disease
DBP	diastolic blood pressure
DHA	docosahexaenoic acid
ECW	extracellular water
EPA	eicosapentaenoic acid
FFM	fat-free mass
FMD	flow-mediated dilation
HR	heart rate
ICW	intracellular water
IF	interstitial fluid
LDF	laser Doppler flowmetry
LTH	local thermal hyperemia
MAP	mean arterial pressure
NO	nitric oxide
NMTG-MD	nitroglycerine-mediated dilation
PF	plasma fluid
PORH	post-occlusive reactive hyperemia
PU	perfusion units
PUFAs	polyunsaturated fatty acids
ROS	reactive oxygen species
SBP	systolic blood pressure
SNPID	sodium nitroprusside-induced
TBW	total body water
WHR	waist-to-hip ratio

## References

[1] Du Z., Qin Y. (2023). Dyslipidemia and Cardiovascular Disease: Current Knowledge, Existing Challenges, and New Opportunities for Management Strategies. J. Clin. Med..

[2] Li Y., Cao G., Jing W., Liu J., Liu M. (2023). Global trends and regional differences in incidence and mortality of cardiovascular disease, 1990−2019: Findings from 2019 global burden of disease study. Eur. J. Prev. Cardiol..

[3] Hadi H.A.R., Carr C.S., Al Suwaidi J. (2005). Endothelial dysfunction: Cardiovascular risk factors, therapy, and outcome. Vasc. Health Risk Manag..

[4] Rippe J.M. (2019). Lifestyle Strategies for Risk Factor Reduction, Prevention, and Treatment of Cardiovascular Disease. Am. J. Lifestyle Med..

[5] Fekete M., Lehoczki A., Kryczyk-Poprawa A., Zábó V., Varga J.T., Bálint M., Fazekas-Pongor V., Csípő T., Rząsa-Duran E., Varga P. (2025). Functional Foods in Modern Nutrition Science: Mechanisms, Evidence, and Public Health Implications. Nutrients.

[6] Oppedisano F., Macrì R., Gliozzi M., Musolino V., Carresi C., Maiuolo J., Bosco F., Nucera S., Caterina Zito M., Guarnieri L. (2020). The Anti-Inflammatory and Antioxidant Properties of n-3 PUFAs: Their Role in Cardiovascular Protection. Biomedicines.

[7] Nađ T., Kolobarić N., Mihaljević Z., Drenjančević I., Šušnjara P., Stupin A., Kardum D., Kralik Z., Kralik G., Košević M. (2025). Effect of n-3 Polyunsaturated Fatty Acids Enriched Chicken Meat Consumption in Relation to Oxidative Stress Marker Levels in Young Healthy Individuals: A Randomized Double-Blind Study. Antioxidants.

[8] Stupin A., Mihalj M., Kolobarić N., Šušnjara P., Kolar L., Mihaljević Z., Matić A., Stupin M., Jukić I., Kralik Z. (2020). Anti-Inflammatory Potential of n-3 Polyunsaturated Fatty Acids Enriched Hen Eggs Consumption in Improving Microvascular Endothelial Function of Healthy Individuals—Clinical Trial. Int. J. Mol. Sci..

[9] Ćurić Ž.B., Masle A.M., Kibel A., Selthofer-Relatić K., Stupin A., Mihaljević Z., Jukić I., Stupin M., Matić A., Kozina N. (2021). Effects of n-3 Polyunsaturated Fatty Acid-Enriched Hen Egg Consumption on the Inflammatory Biomarkers and Microvascular Function in Patients with Acute and Chronic Coronary Syndrome—A Randomized Study. Biology.

[10] Spite M., Clària J., Serhan C.N. (2014). Resolvins, Specialized Proresolving Lipid Mediators, and Their Potential Roles in Metabolic Diseases. Cell. Metab..

[11] Mihalj M., Stupin A., Kolobarić N., Tartaro Bujak I., Matić A., Kralik Z., Jukić I., Stupin M., Drenjančević I. (2020). Leukocyte Activation and Antioxidative Defense Are Interrelated and Moderately Modified by n-3 Polyunsaturated Fatty Acid-Enriched Eggs Consumption—Double-Blind Controlled Randomized Clinical Study. Nutrients.

[12] Versari D., Daghini E., Virdis A., Ghiadoni L., Taddei S. (2009). Endothelial Dysfunction as a Target for Prevention of Cardiovascular Disease. Diabetes Care.

[13] Zehr K.R., Walker M.K. (2018). Omega-3 polyunsaturated fatty acids improve endothelial function in humans at risk for atherosclerosis: A review. Prostaglandins Other Lipid Mediat..

[14] Canoy D., Bundred P. (2011). Obesity in children. BMJ Clin. Evid..

[15] DeMers D., Wachs D. (2022). Physiology, Mean Arterial Pressure. StatPearls [Internet].

[16] Roustit M., Cracowski J.C. (2012). Non-invasive assessment of skin microvascular function in humans: An insight into methods. Microcirculation.

[17] Kos M., Nađ T., Stupin A., Drenjančević I., Kolobarić N., Šušnjara P., Mihaljević Z., Damašek M., Pušeljić S., Jukić I. (2024). Juvenile primary hypertension is associated with attenuated macro- and microvascular dilator function independently of body weight. J. Hypertens..

[18] Colussi G., Catena C., Sechi A.L. (2015). Omega-3 Polyunsaturated Fatty Acids Effects on the Cardiometabolic Syndrome and their Role in Cardiovascular Disease Prevention: An Update from the Recent Literature. Recent Adv. Cardiovasc. Drug Discov..

[19] Yokoyama M., Origasa H., Matsuzaki M., Matsuzawa Y., Saito Y., Ishikawa Y., Oikawa S., Sasaki J., Hishida H., Itakura H. (2007). Effects of eicosapentaenoic acid on major coronary events in hypercholesterolaemic patients (JELIS): A randomised open-label, blinded endpoint analysis. Lancet.

[20] Bhatt D.L., Steg P.G., Miller M., Brinton E.A., Jacobson T.A., Ketchum S.B., Doyle R.T., Juliano R.A., Jiao L., Granowitz C. (2019). Cardiovascular Risk Reduction with Icosapent Ethyl for Hypertriglyceridemia. N. Engl. J. Med..

[21] Mulrow C.D. (1994). Diet supplementation with fish oils and blood pressure reduction: A meta-analysis. ACP J. Club.

[22] Knapp H.R., FitzGerald G.A. (1989). The Antihypertensive Effects of Fish Oil. N. Engl. J. Med..

[23] Bønaa K.H., Bjerve K.S., Straume B., Gram I.T., Thelle D. (1990). Effect of Eicosapentaenoic and Docosahexaenoic Acids on Blood Pressure in Hypertension. N. Engl. J. Med..

[24] Kestin M., Clifton P., Belling G., Nestel P. (1990). n-3 fatty acids of marine origin lower systolic blood pressure and triglycerides but raise LDL cholesterol compared with n-3 and n-6 fatty acids from plants. Am. J. Clin. Nutr..

[25] Rasmussen B.M., Vessby B., Uusitupa M., Berglund L., Pedersen E., Riccardi G., Rivellese A.A., Tapsell L., Hermansen K. (2006). Effects of dietary saturated, monounsaturated, and n−3 fatty acids on blood pressure in healthy subjects. Am. J. Clin. Nutr..

[26] Stupin A., Rasic L., Matic A., Stupin M., Kralik Z., Kralik G., Grcevic M., Drenjancevic I. (2018). Omega-3 polyunsaturated fatty acids-enriched hen eggs consumption enhances microvascular reactivity in young healthy individuals. Appl. Physiol. Nutr. Metab..

[27] Oh S., Ryue J., Hsieh C., Bell D. (1991). Eggs enriched in ω-3 fatty acids and alterations in lipid concentrations in plasma and lipoproteins and in blood pressure. Am. J. Clin. Nutr..

[28] Bender N., Portmann M., Heg Z., Hofmann K., Zwahlen M., Egger M. (2014). Fish or n3-PUFA intake and body composition: A systematic review and meta-analysis. Obes. Rev..

[29] Zhang Y.Y., Liu W., Zhao T.Y., Tian H.M. (2017). Efficacy of omega-3 polyunsaturated fatty acids supplementation in managing overweight and obesity: A meta-analysis of randomized clinical trials. J. Nutr. Health Aging.

[30] Du S., Jin J., Fang W., Su Q. (2015). Does Fish Oil Have an Anti-Obesity Effect in Overweight/Obese Adults? A Meta-Analysis of Randomized Controlled Trials. PLoS ONE.

[31] Monnard C.R., Dulloo A.G. (2021). Polyunsaturated fatty acids as modulators of fat mass and lean mass in human body composition regulation and cardiometabolic health. Obes. Rev..

[32] Tomczyk M. (2024). Omega-3 Fatty Acids and Muscle Strength—Current State of Knowledge and Future Perspectives. Nutrients.

[33] Khan F. (2003). The effects of dietary fatty acid supplementation on endothelial function and vascular tone in healthy subjects. Cardiovasc. Res..

[34] Daci A., Celik Z., Ozen G., Dashwood M., Dogan B.S.U., Topal G. (2020). Effect of omega-3 polyunsaturated fatty acids in modulation of vascular tone under physiological and pathological conditions. Eur. J. Pharm. Sci..

[35] Stirban A., Nandrean S., Götting C., Tamler R., Pop A., Negrean M., Gawlowski T., Stratmann B., Tschoepe D. (2010). Effects of n–3 fatty acids on macro- and microvascular function in subjects with type 2 diabetes mellitus. Am. J. Clin. Nutr..

[36] Arabi S.M., Bahari H., Chambari M., Bahrami L.S., Mohaildeen Gubari M.I., Watts G.F., Sahebkar A. (2024). Omega-3 fatty acids and endothelial function: A GRADE-assessed systematic review and meta-analysis. Eur. J. Clin. Investig..

[37] Colussi G., Catena C., Novello M., Bertin N., Sechi L.A. (2017). Impact of omega-3 polyunsaturated fatty acids on vascular function and blood pressure: Relevance for cardiovascular outcomes. Nutr. Metab. Cardiovasc. Dis..

[38] Wang Q., Liang X., Wang L., Lu X., Huang J., Cao J., Li H., Gu D. (2012). Effect of omega-3 fatty acids supplementation on endothelial function: A meta-analysis of randomized controlled trials. Atherosclerosis.

[39] Du Y., Taylor C.G., Zahradka P. (2019). Modulation of endothelial cell responses and vascular function by dietary fatty acids. Nutr. Rev..

[40] Karimi E., Keske M.A., Beba M., Kaur G. (2025). Effects of omega-3 fatty acids on skeletal muscle vascular health and metabolism. Curr. Opin. Clin. Nutr. Metab. Care.

[41] Calder P.C. (2015). Marine omega-3 fatty acids and inflammatory processes: Effects, mechanisms and clinical relevance. Biochim. Biophys. Acta Mol. Cell. Biol. Lipids.

